# FACEmemory^®^, an Innovative Self-Administered Online Memory Assessment Tool

**DOI:** 10.3390/jcm13237274

**Published:** 2024-11-29

**Authors:** Montserrat Alegret, Josep Blazquez-Folch, Alba Pérez, Gemma Ortega, Ana Espinosa, Nathalia Muñoz, Angela Sanabria, Fernando García-Gutiérrez, Emilio Alarcon-Martin, Maitee Rosende-Roca, Liliana Vargas, Juan Pablo Tartari, Dorene M. Rentz, Sergi Valero, Agustín Ruiz, Mercè Boada, Marta Marquié

**Affiliations:** 1 Ace Alzheimer Center Barcelona, Universitat Internacional de Catalunya, 08028 Barcelona, Spain; jblazquez@fundacioace.org (J.B.-F.); aperez@fundacioace.org (A.P.); gortega@fundacioace.org (G.O.); aespinosa@fundacioace.org (A.E.); nmunoz@fundacioace.org (N.M.); asanabria@fundacioace.org (A.S.); fgarcia@fundacioace.org (F.G.-G.); ealarcon@fundacioace.org (E.A.-M.); mrosende@fundacioace.org (M.R.-R.); lvargas@fundacioace.org (L.V.); jptartari@fundacioace.org (J.P.T.); svalero@fundacioace.org (S.V.); aruiz@fundacioace.org (A.R.); mboada@fundacioace.org (M.B.); mmarquie@fundacioace.org (M.M.); 2 Networking Research Center on Neurodegenerative Diseases (CIBERNED), Instituto de Salud Carlos III, 28029 Madrid, Spain; 3 Center for Alzheimer Research and Treatment, Department of Neurology, Brigham and Women’s Hospital, Boston, MA 02115, USA; drentz@bwh.harvard.edu; 4 Department of Neurology, Massachusetts General Hospital, Boston, MA 02114, USA

**Keywords:** memory, Alzheimer’s disease, early detection, mild cognitive impairment, new technologies, digital biomarkers

## Abstract

**Background**: Alzheimer’s disease (AD) dementia and mild cognitive impairment (MCI) are currently underdiagnosed in the community, and early detection of cognitive deficits is crucial for timely intervention. FACEmemory^®^, the first completely self-administered online memory test with voice recognition, has been launched as an accessible tool to detect such deficits. This study aims to investigate the neuropsychological associations between FACEmemory subscores and cognitive composites derived from traditional paper-and-pencil neuropsychological tests and to develop an optimal algorithm using FACEmemory data and demographics to discriminate cognitively healthy (CH) individuals from those with MCI. **Methods**: A total of 669 participants (266 CH, 206 non-amnestic MCI [naMCI], and 197 amnestic MCI [aMCI]) were included. Multiple linear regression analyses were conducted using a cognitive composite as the dependent variable and FACEmemory subscores and demographic data (age, sex, and schooling) as independent variables. Machine learning models were compared to identify an optimal algorithm for distinguishing between CH and MCI (whole MCI, aMCI, and naMCI). **Results**: Multiple regression analyses showed associations between FACEmemory scores and the domains of memory (ρ = 0.67), executive functions (ρ = 0.63), visuospatial/visuoperceptual abilities (ρ = 0.55), language (ρ = 0.43), praxis (ρ = 0.52), and attention (ρ = 0.31). An optimal algorithm distinguished between CH and aMCI, achieving a FACEmemory cutoff score of 44.5, with sensitivity and specificity values of 0.81 and 0.72, respectively. **Conclusions**: FACEmemory is a promising online tool for identifying early cognitive impairment, particularly aMCI. It may contribute to addressing the underdiagnosis of MCI and dementia in the community and in promoting preventive strategies.

## 1. Introduction

Alzheimer’s disease (AD) is the most common form of neurodegenerative dementia, typically beginning with subtle memory deficits [1]. Unfortunately, AD remains underdiagnosed in the community [2], despite the identification of 14 modifiable risk factors for developing dementia (low education, hypertension, high LDL cholesterol, diabetes, obesity, smoking, excessive alcohol consumption, depression, physical inactivity, traumatic brain injury, air pollution, hearing and visual loss, and social isolation) [3,4]. Nearly 45% of dementia cases could potentially be prevented with effective prevention policies [3,4]. Furthermore, several disease-modifying therapies for AD (e.g., monoclonal antibodies against beta-amyloid) have demonstrated amyloid clearance from the brain and modest cognitive decline slowdown, recently gaining approval for clinical use worldwide [5]. In this context, detecting the early symptoms of AD, such as memory loss or executive function deficits, is crucial for enabling timely intervention and management strategies that could slow cognitive decline progression and enhance patients’ quality of life.

In response to the growing need for early cognitive impairment detection and the increasing interest in new technologies, our team at Ace Alzheimer Center Barcelona developed the online FACEmemory^®^ platform [6]. Originally released as an in-person test with minimal supervision in 2015 [7], a fully optimized online version became freely available to the community in 2021 [6]. FACEmemory is the first completely self-administered memory test with voice recognition and automatic scoring [6]. Testing at Ace Alzheimer Center Barcelona, with minimal supervision, demonstrated the reliability of FACEmemory’s automated scoring in detecting mild cognitive impairment (MCI), particularly the amnestic type [7], which is at higher risk of progression to AD dementia [8]. Moreover, FACEmemory scores have shown associations with AD phenotype and biomarkers in both early- and late-onset cases [7,9].

Computerized tests offer numerous advantages over traditional paper-and-pencil assessments, including standardized administration, accurate scoring, cost-effectiveness, and improved accessibility [10]. A systematic comparison of computerized cognitive tools, including FACEmemory with minimal supervision [7], found that memory-sensitive tools (e.g., FACEmemory, MemTrax test) and those assessing executive functions (e.g., Instrumented Trail Making Test, Tablet-based Cancellation Test) were particularly effective in detecting cognitive impairment [10]. Moreover, the online version of FACEmemory stands out as the first fully self-administered associative memory test to include learning, long-term free recall, and recognition tasks with voice recognition, eliminating the need for supervision by a healthcare professional [6].

Designed to address the underdiagnosis of cognitive impairment and AD [2], the online FACEmemory platform reached over 3000 adults from 37 countries in its first 1.5 years, with 82.1% of users reporting memory concerns [6], a known risk factor for AD in individuals over 60 [11]. At our center, we observed the benefits of an open house initiative (a screening approach to facilitate memory assessments for individuals over 50 without requiring physician visits) which successfully engaged individuals with subjective cognitive decline (SCD) [12] and MCI [13]. FACEmemory, adapted from the FNAME-12 [14] (an abbreviated version of the Face–Name Associative Memory Exam (FNAME) [15]), offers a promising pre-screening tool for individuals with SCD and MCI, though it may be too challenging for patients with dementia, who usually receive minimal scores [14].

According to the theory that various cognitive processes contribute to different memory stages [16], a machine learning (ML) analysis revealed that FACEmemory subscores distinguish four memory patterns: preserved execution, storage, dysexecutive, and global memory impairments [6]. These findings suggest that FACEmemory can support the early detection of cognitive impairment in individuals over 50, enhancing conventional clinical assessments.

This study aims to compare data from the online FACEmemory platform with results from formal cognitive assessments at a memory clinic to facilitate earlier MCI identification. Specifically, the main objectives of this study are (1) to investigate the neuropsychological associations between FACEmemory subscores and cognitive composites derived from traditional paper-and-pencil neuropsychological tests and (2) to develop an optimal algorithm using FACEmemory subscores and demographic data to discriminate cognitively healthy (CH) individuals from those with MCI.

## 2. Materials and Methods

### 2.1. Participants

This study included individuals over the age of 50 who completed the online FACEmemory platform and underwent diagnostic evaluation at Ace Alzheimer Center Barcelona Memory Unit within six months, with either CH or MCI diagnoses from June 2021 to May 2024. Individuals diagnosed with dementia, based on impairment in activities of daily living, or with severe auditory or visual impairments were excluded. Demographic information, including age, sex, and years of formal education, was collected from all participants.

### 2.2. The FACEmemory^®^ Platform

As previously described [6], the FACEmemory^®^ platform is available free of charge for individuals interested in memory assessment. The platform can be used on a tablet or computer with voice recognition and an internet connection. Participants were encouraged to complete FACEmemory independently at home; however, those without access to electronic devices performed the test independently at the Ace Alzheimer Center Barcelona’s Memory Unit.

Upon accessing FACEmemory, users select their preferred language (Spanish or Catalan), accept informed consent, and provide demographic information (age, sex, education level, country of origin) and an email address to receive results. Users are asked about subjective memory complaints and associated concerns with three questions: (1) “Do you feel that your memory has worsened?” (yes/no), (2) “Are you worried about it?” (yes/no), and (3) “Since when have you noticed it?” (in years). Then, FACEmemory is introduced through a brief video, followed by an audio test to ensure optimal voice recognition.

FACEmemory includes two learning trials: a short-term memory task and a long-term memory task involving face, name, and occupation recognition. In each learning trial (Learning 1 and Learning 2), 12 faces associated with a name and an occupation are shown for 8 s. The sequence is changed between trials. Users are instructed to read each name and occupation aloud and to try to remember. Then, the application prompts users to press the red microphone button and say the name and occupation associated with each face they remember.

A short-term memory task (Short-term) begins two minutes after the second learning trial. The long-term memory assessment (Long-term), including free recall and recognition tasks, starts 15 min after the second learning trial. First, users are asked to select from three faces displayed on-screen the one shown during the learning trials (Face Recognition). After choosing a face, the correct face is displayed, and users are prompted to say the name and occupation associated with each face. After providing their answer, a screen appears with the correct face and two rows below it, each containing 3 name and 3 occupation options. The users are instructed to select the name and occupation that they recall being associated with the face (Recognitions).

After the short-term memory test, users can complete an optional medical and family history questionnaire (Open House Initiative [OHI] Questionnaire) [12], but completion is not mandatory. If users do not complete the questionnaire within the assigned time, the system proceeds automatically to long-term recall tasks.

All subscores range from 0 to 12, and the total FACEmemory ranges from 0 to 132. As reported previously [7], the sum of all free recalled subscores, excluding Face Recognition and Recognitions (FR, REN, and REO), was also calculated on a 0 to 96 scale. Test completion time (in minutes) was recorded, and the derived subscores, including consecutive failures and omissions, were calculated.

Once completed, users cannot retake the test for one year, receiving a new invitation 11 months later with an alternate version (A or B) to minimize practice effects.

### 2.3. Diagnosis Evaluation

All participants underwent diagnostic evaluation at the Ace Alzheimer Center Barcelona Memory Clinic, which included a social worker interview, a neurological examination, and a complete neuropsychological assessment using the Neuropsychological Battery of Ace (NBACE©), which includes normative data [17] and impairment cut-offs [18]. NBACE assesses cognitive domains such as information processing speed, orientation, attention, verbal learning and long-term memory, language, praxis, and visuospatial, visuoperceptual, and executive functions [17].

Clinical diagnosis for CH individuals, with or without subjective memory complaints, required the absence of objective cognitive impairment, preserved NBACE scores [17,18], a Mini-Mental State Examination (MMSE) score ≥ 27 [19,20], a Clinical Dementia Rating (CDR) [21] score of 0, and no functional impairment, with a score below 4 on the Blessed Dementia Rating Scale (BDRS) [22,23]. Diagnosis for MCI patients included subjective cognitive complaints, preserved global cognition (MMSE score ≥ 24), normal performance in activities of daily living (BDRS score < 4), a CDR score of 0.5, and measurable impairment in memory and/or another cognitive function (amnestic MCI [aMCI] or non-amnestic MCI [naMCI]) [8,24].

### 2.4. Statistical and Descriptive Analyses

Statistical analyses were performed using Statistical Package for the Social Sciences version 26 (SPSS Inc., Chicago, IL, USA) and Python version 3.11.7. Data were examined for normality, skewness, and range restriction.

Descriptive analyses used *t*-tests and chi-square tests to compare FACEmemory, demographic, clinical, memory complaints, and medical history variables between CH and MCI groups.

Multiple linear regression analyses were conducted using a cognitive composite as the dependent variable, with age, sex, education, and FACEmemory subscores as independent variables. The analyses included models for attention, executive functions, language, memory, reality orientation, praxis, and visuospatial/visuoperceptual cognitive composites, as detailed in Appendix A. Neuropsychological composites were estimated through structural equation modeling (SEM), guided by exploratory factor analysis and expert consensus. Seven composites were analyzed: memory, attention, visuospatial/visuoperception, executive functions, language, reality orientation, and praxis. The memory composite was derived from the long-term and recognition memory variables of the Word List subtest of the Wechsler Memory Scale, Third Edition (WMS-III). Attention function was assessed using the Digit Forward and Digit Backward subtests of the Wechsler Adult Intelligence Scale, Third Edition (WAIS-III). The visuospatial/visuoperceptive composite included the 15-Objects Test, Poppelreuter-type overlap figures, and Luria’s Clock Test. The executive function composite was constructed from Phonetic and Semantic Verbal Fluencies and the Automatic Inhibition subtest of the Syndrom Kurtz Test (SKT). The language composite included the abbreviated Boston Naming Test (15-BNT) and the Verbal Comprehension and Repetition tests. The reality orientation composite was calculated using the Temporal, Spatial, and Personal orientation tests. Finally, the praxis composite was based on the Imitation, Ideomotor, and Block Design tests.

The SEM models were fitted using robust maximum likelihood estimation, except for the reality orientation and praxis composites, which were calculated using a weighted least square mean and variance-adjusted estimator due to their ordinal distribution. The variances of the latent variables were fixed at 1 for model identification [25]. The R version 4.3.3 package lavaan version 0.6–18 was used to calculate the composites [26].

All effects were considered significant at a threshold of *p* < 0.05, and all hypotheses were tested bidirectionally at a 95% confidence level.

For the multiple regression model with 15 predictors, assuming a statistical power of 80%, a significance level of 0.05, and a large effect size (\(f^2 = 0.35\)), the required sample size was calculated to be at least 167 participants [27].

### 2.5. FACEmemory for Classification Between CH and MCI

To develop an algorithm capable of distinguishing between CH and MCI groups (including all MCI, aMCI, and naMCI), ML techniques were applied using the FACEmemory variables. Additionally, demographic information (age, sex, and education) was included as input variables to compare the performance of ML models trained solely on FACEmemory data with those taught on a combination of FACEmemory and demographic data.

For the classification tasks (CH-MCI, CH-aMCI, and CH-naMCI), the following ML models were utilized: k-nearest neighbor (KNN), decision tree (DT), support vector machine (SVM), random forest (RF), and extreme gradient boosting (XGB). Cross-validation (CV) with class stratification ensured balanced representation across groups. Model performance was assessed using sensitivity, specificity, and balanced accuracy (BA). Input variables were standardized to z-scores based on the training data statistics.

Hyperparameter optimization (HPO) was performed with a nested CV framework, using predefined search spaces detailed in Appendix B. Table A8, Table A9, Table A10, Table A11 and Table A12 in Appendix B provide the specific hyperparameter configurations for the KNN, DT, SVM, RF, and XGB models, respectively. Outer CV consisted of five folds, while inner CV also used five folds for HPO. Nested CV models were used to predict test sets and final performance metrics were calculated as the average of these predictions. HPO was implemented using the open-source Optuna library version 3.6.0 [28], which employed Bayesian optimization (BO) with a tree-structured Parzen estimator (TPE) as a surrogate model [29].

ML model performance was evaluated against a baseline cutoff model, which differentiated diagnostic groups using a threshold on the FACEmemory total score. This threshold was optimized to maximize BA.

All models were implemented in Python. The scikit-learn library version 1.2.2 was used for the RF, KNN, and SVM algorithms [30], and the xgboost package version 2.0.3 was employed for XGB models [31].

Additionally, models were trained using FACEmemory subscores for data up to specific test blocks (Learning 1, Learning 2, Short-term, Long-term, Face Recognition, and Recognitions). This approach allowed models to classify diagnostic groups based on incomplete test data, providing insights into whether the test duration could be shortened or was already optimal. This methodology also allowed for the generation of results for those individuals who could not complete the entire test for any reason. While this feature selection approach does not have a widely standardized name, it shares similarities with Blockwise Feature Selection [32].

## 3. Results

### 3.1. Descriptive

A total of 669 individuals (251 men and 418 women) participated in this study. The mean age was 68.4 years (standard deviation [SD]: 8.7), with ages ranging from 50 to 93 years. Most participants (98.1%) had at least six years of formal education, with 58% having completed elementary or high school, 40.1% holding a university degree, and only 1.9% with less than an elementary school education. A majority (62.6%) completed the FACEmemory test in Spanish (419 participants), while 250 participants completed it in Catalan.

Most participants (94.5%) reported subjective memory complaints with associated concerns. After the clinical evaluation at the Ace Alzheimer Center Barcelona Memory Clinic, 266 participants were classified as CH, while 403 were diagnosed with MCI, including 206 with naMCI and 197 with aMCI. The MCI group was significantly older, had less formal education, and showed lower MMSE scores than the CH group. However, the mean MMSE scores for CH and MCI groups were 29.3 and 28.2, respectively (for details, see Table 1). Compared to CH participants, the MCI group had a lower frequency of individuals who fully completed the OHI Questionnaire and reported visual abnormalities and a higher frequency of participants who completed the test in Spanish (Table 1). The average time between completing the FACEmemory test and the diagnostic evaluation was 5.2 days (SD: 54.0).

**Figure 1:**
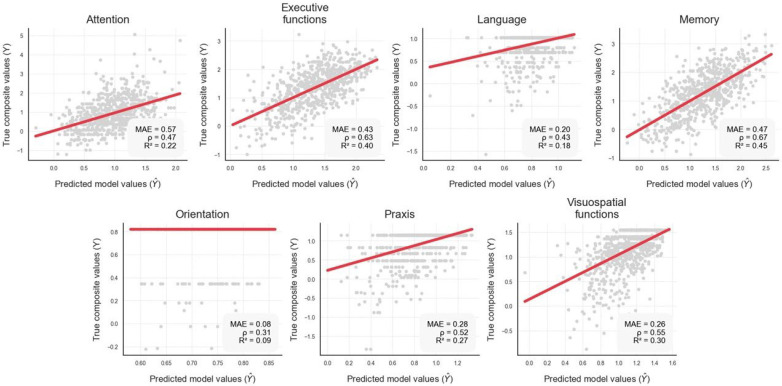
Correlation between predicted values generated by multiple regression analysis models and the actual values for each cognitive domain analyzed. MAE: mean absolute error; ρ: correlation between model predictions (*Ŷ*) and true values (*Y*); R2: coefficient of determination.

As detailed in Table 2, the MCI group performed significantly worse on FACEmemory variables than the CH group, with a mean difference of 21 points in the total score. Table 3 shows a progressive decline in performance from CH to naMCI and aMCI groups. Multiple regression analyses revealed associations between FACEmemory scores and the cognitive domains of the NBACE, including memory (ρ = 0.67), executive functions (ρ = 0.63), visuospatial/visuoperceptual (ρ = 0.55), language (ρ = 0.43), praxis (ρ = 0.52), and attention (ρ = 0.31) (Figure 1). For further details, see Appendix A (Table A1, Table A2, Table A3, Table A4, Table A5, Table A6 and Table A7).

### 3.2. FACEmemory for the Discrimination Between MCI Subgroups

Regarding the MCI subgroups, the aMCI and naMCI groups showed no significant differences in age, sex, or years of schooling. However, the aMCI group performed significantly worse on both the MMSE and FACEmemory, with average scores that were 1 point and 12 points lower, respectively (Table 3).

To discriminate between CH and MCI (including all MCI, naMCI, and aMCI), the model with the highest BA was selected.

The best performance for distinguishing between CH and aMCI was achieved for the Long-term block. In contrast, the most effective approach for differentiating CH from naMCI involved using data up to Face Recognition with an RF model incorporating subscores and demographic features. While adding demographic information did not improve the CH and aMCI classification, it did enhance the CH and naMCI classification. For the Learning 2 block, the CH-aMCI classification reached a BA of 0.74, while the Long-term block achieved the highest BA of 0.76. The model using the cumulative score up to the Long-term block performed best in distinguishing CH from aMCI (Table 4).

**Figure 2:**
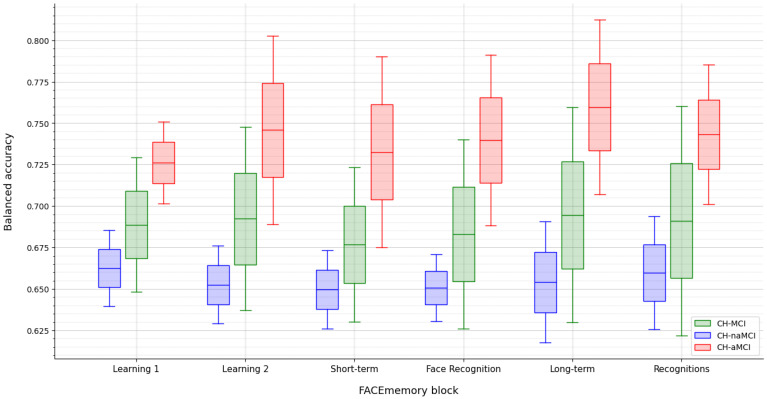
Performance of ML models on the test set across varying FACEmemory assessment lengths. The *x*-axis represents each FACEmemory block, where each block includes its own subscores variables along with cumulative data from all preceding blocks. CH: cognitively healthy; MCI: mild cognitive impairment; naMCI; non-amnestic mild cognitive impairment; aMCI: amnestic mild cognitive impairment.

To maximize BA, a total FACEmemory cutoff between CH and aMCI was established at 44.5, achieving sensitivity and specificity values of 0.81 and 0.72, respectively (see Table 4 and Figure 2). Without the Face Recognition and Recognition blocks, a cutoff of 33.5 yielded sensitivity and specificity values of 0.82 and 0.70, respectively.

## 4. Discussion

The findings of this study suggest that FACEmemory is a promising online tool for identifying early cognitive impairment, particularly aMCI, within the community and tracking cognitive progression from normal aging to aMCI. Significant associations were between FACEmemory subscores and cognitive composites obtained from traditional paper-and-pencil neuropsychological tests, validating FACEmemory as an effective digital test of memory and related cognitive domains. Building on this validation, the development and application of machine learning algorithms achieved sensitivity and specificity values that enable the distinction between CH and MCI, particularly in detecting amnestic MCI. This tool has the potential to identify cognitive impairments, including aMCI and dementia, particularly within the community. It offers a valuable opportunity to address the current underdiagnosis of cognitive impairment and AD [2]. The innovation in this study lies in the optimized web-based version of FACEmemory, a complex memory test that covers all memory processes, including learning, long-term memory, and recognition. Importantly, it allows individuals to complete the test independently at home without the need for specialist supervision [6].

FACEmemory is derived from FNAME-12 [14], a cognitive test with 12 face–name–occupation associations developed to identify the early stages of AD, including preclinical and prodromal phases, and to track cognitive progression [14]. Consistent with previous findings [7,14], performance on the online FACEmemory worsened progressively from CH individuals to those with naMCI and aMCI, reflecting a gradual decline in complex memory functions from normal aging to aMCI, which carries an increased risk of progression to dementia, primarily AD [8]. Although the effects of age, education, and sex may vary, our results align with prior reports linking worse associative memory performance on the original FNAME-12 and computerized FACEmemory to lower education levels and older age, but not to sex [9,14].

FACEmemory subscores showed links across all cognitive domains, with strong associations with memory and executive function domains, moderate associations with the visuospatial/visuoperceptual, language, and praxis domains, and a weaker association with attention. These findings suggest that FACEmemory scoring relates not only to memory but also to other cognitive domains, particularly executive functions, supporting the notion that distinct cognitive processes contribute to different stages of memory execution [16]. Furthermore, given the complexity of FACEmemory as a self-administered episodic memory test, successful performance requires optimal functioning across multiple cognitive domains [6], leading to scores below the impairment cutoff in individuals with MCI [7].

A recent systematic review of digital cognitive biomarkers from computerized tests for detecting MCI and dementia, including the FACEmemory with minimal supervision [7], found that digital biomarkers related to memory and executive functions were more sensitive than those related to other cognitive domains [10]. Thus, our findings further support FACEmemory’s potential as a valuable tool for detecting cognitive impairment and even very mild MCI (less severe than MCI), as noted by Dr. Frank Jessen [11].

Comparisons of ML models identified the best algorithm for distinguishing between CH and MCI groups (including all MCI, aMCI, and naMCI) based on cumulative FACEmemory subscores, both with and without demographic information. The best performance was achieved using long-term memory scores, particularly for CH vs. aMCI classification. Although lower FACEmemory scores were associated with lower education levels and older age, adding demographic information did not enhance the CH-aMCI classification. Given FACEmemory’s focus on episodic memory, these results underscore the importance of including long-term memory tasks, despite the additional 15 min they require. These findings confirm the optimal test duration as originally designed [7,14].

Additionally, the baseline model, which used a FACEmemory cutoff score, performed best for distinguishing CH from aMCI. A cutoff score of 44.5 points (up to the Long-term block) yielded sensitivity and specificity values of 0.81 and 0.72, respectively. Our previous study reported a cutoff score of 31.5 (without recognition tasks) for CH and aMCI classification [7]. In this study, the cutoff score without recognition tasks was 33.5, yielding sensitivity and specificity values of 0.82 and 0.70, respectively.

An intriguing result from the ML analyses was that models trained on data from just the two face–name learning trials (Learning 1 and 2) and those trained up to the long-term memory task (Long-term) demonstrated similar performance in distinguishing CH and MCI as the baseline models. This finding supports the platform’s potential to provide accurate results even for individuals unable to complete the test in full for any reason.

We acknowledge several limitations in this study. First, although FACEmemory was offered at no cost to the community, the data analyzed were limited to individuals evaluated at the Ace Alzheimer Center Barcelona’s Memory Clinic. This restriction confines this study to a single center, but the findings could potentially be generalizable to other populations, including English speakers, as FACEmemory is also available in English (https://www.fundacioace.com/en/check-your-memory-online-with-facememory.html (accessed on 26 November 2024)). Second, we cannot rule out that some CH individuals with low FACEmemory scores might have preclinical AD. However, AD-related biomarkers are not yet approved for clinical use in asymptomatic aging populations. Third, this is a cross-sectional study, so longitudinal research is needed to determine whether low baseline FACEmemory scores are associated with an increased risk of developing dementia or AD and to track whether individuals who progress to dementia exhibit declining FACEmemory scores over time, while non-converters maintain stable scores. Fourth, participants completed the test independently, but we cannot guarantee that the test was completed without any external assistance. Additionally, we believe it would be unethical to record the test execution. Finally, because our objective was to detect MCI and AD, only participants over 50 were included. However, FACEmemory could also be valuable for identifying cognitive impairment in younger populations, such as in high schools and college settings, where it could serve as an effective memory test.

## 5. Conclusions

FACEmemory is a promising online tool for identifying early cognitive impairment, particularly aMCI, within the community and tracking cognitive progression from normal aging to aMCI. It provides individuals concerned about their memory with the option to complete this prescreening memory test comfortably at home, using an electronic device and an internet connection, without the need for a healthcare specialist’s supervision. FACEmemory may contribute to addressing the underdiagnosis of MCI and dementia in the community and to promoting preventive strategies.

## Figures and Tables

**Table 1:** Demographic, clinical, memory complaints, and medical history variables for the whole study sample, with a comparison between CH and MCI groups.

	Whole Sample	CH	MCI	Statistics
**n**	669	266	403	
Age (mean, SD)	68.41 (8.78)	66.48 (7.95)	69.68 (9.07)	−4.68 (1) *
Sex (n woman, % woman; n man, % man)	418 (62.48) 251 (37.52)	166 (62.41) 100 (37.59)	252 (62.53) 151 (37.47)	0.0 (2)
Years of schooling (mean, SD)	11.91 (4.40)	13.06 (4.07)	11.15 (4.45)	5.62 (1) *
Level of schooling (N, %)				18.14 (2) *
Less than elementary school	13 (1.94)	1 (0.38)	12 (2.98)	
Elementary or high school	388 (58.00)	135 (50.75)	253 (62.78)	
University degree	268 (40.06)	130 (48.87)	138 (34.24)	
MMSE (mean, SD)	28.63 (1.41)	29.31 (0.88)	28.19 (1.51)	10.73 (1) *
Language (n Spanish, % Spanish; n Catalan, % Catalan)	419 (62.63) 250 (37.37)	148 (55.64) 118 (44.36)	271 (67.25) 131 (32.75)	10.16 (2) *
Memory complaints (n, %)	632 (94.47)	247 (92.86)	385 (95.53)	1.71 (2)
OHI questionnaire completed (n, %)	257 (38.42)	151 (56.77)	106 (26.30)	61.58 (2) *
Auditory abnormalities (n, %)	199 (29.75)	78 (29.32)	121 (30.02)	0.01 (2)
Visual abnormalities (n, %)	261 (39.01)	118 (44.36)	143 (35.48)	4.94 (2) *
Neurologic/psychiatric disease (n, %)	213 (31.84)	76 (28.57)	137 (34.00)	1.93 (2)

CH: cognitively healthy; MCI: mild cognitive impairment; MMSE: Mini-Mental State Examination; OHI: open house initiative; SD: standard deviation; (1): *t*-test; (2): χ2-test; * *p*-value < 0.05.

**Table 2:** Performance on FACEmemory subscores and total score for the whole sample and by cognitive groups.

FACEmemory Block	Variable (Min–Max)	Whole Sample (Mean, SD)	CH (Mean, SD)	MCI (Mean, SD)	Statistics (1)
Learning 1	LN1 (0–12)	1.43 (2.14)	2.23 (2.43)	0.90 (1.73)	8.26 *
	LO1 (0–12)	3.89 (2.74)	5.00 (2.69)	3.16 (2.52)	8.99 *
	CFN (0–6)	5.51 (1.18)	5.17 (1.46)	5.73 (0.89)	6.16 *
	CFO (0–6)	4.11 (1.90)	3.43 (1.99)	4.56 (1.69)	7.88 *
Learning 2	LN2 (0–12)	3.69 (3.27)	5.36 (3.32)	2.59 (2.72)	11.79 *
	LO2 (0–12)	6.25 (3.11)	7.52 (2.79)	5.42 (3.02)	9.07 *
	CFN (0–6)	4.05 (2.05)	3.07 (2.14)	4.69 (1.70)	10.86 *
	CFO (0–6)	2.46 (2.08)	1.71 (1.83)	2.95 (2.09)	7.88 *
Short-term	RSN (0–12)	3.38 (3.27)	5.00 (3.48)	2.31 (2.63)	11.36 *
	RSO (0–12)	6.12 (3.10)	7.41 (2.78)	5.26 (3.00)	9.33 *
	CFN (0–6)	4.36 (2.01)	3.47 (2.22)	4.94 (1.61)	9.91 *
	CFO (0–6)	2.60 (2.08)	1.85 (1.91)	3.09 (2.04)	7.89 *
Face Recognition	FR (0–12)	11.88 (0.49)	11.94 (0.26)	11.84 (0.59)	2.60 *
Long-term	RLN (0–12)	3.17 (3.26)	4.86 (3.45)	2.05 (2.58)	12.03 *
	RLO (0–12)	5.57 (3.28)	7.02 (2.90)	4.62 (3.17)	9.91 *
	CFN (0–6)	4.38 (2.03)	3.40 (2.24)	5.03 (1.58)	11.03 *
	CFO (0–6)	2.96 (2.17)	2.12 (1.94)	3.51 (2.13)	8.55 *
Recognitions	REN (0–12)	8.56 (3.09)	9.97 (2.40)	7.64 (3.14)	10.28 *
	REO (0–12)	11.11 (1.53)	11.62 (0.86)	10.78 (1.76)	7.23 *
	NO (0–18)	2.87 (3.02)	3.35 (3.01)	2.56 (2.99)	3.33 *
	OO (0–23)	5.83 (3.52)	6.03 (3.71)	5.70 (3.40)	1.18
	Total score ** (0–132)	65.06 (22.79)	77.93 (20.50)	56.58 (20.12)	13.33 *
	Execution time (in min)	25.38 (6.47)	23.62 (5.76)	26.53 (6.65)	5.83 *

(1): *t*-test, * *p*-value < 0.05; SD: standard deviation; min-max: minimum–maximum registered; LN1: names recalled in learning 1; LN2: names recalled in learning 2; LO1: occupations recalled in learning 1; LO2: occupations recalled in learning 2; RSN: names in short-term recall; RSO: occupations in short-term recall; RLN: names in long-term recall; RLO: occupations in long-term recall; FR: face recognition; REN: names correctly recognized; REO: occupations correctly recognized; NO: names omissions; OO: occupations omissions; CFN: consecutive name fails in each block; CFO: consecutive occupation fails in each block. ** Total score is the sum of all subscores, including recognition tasks.

**Table 3:** Clinical and sociodemographic characteristics of the MCI group, stratified by amnestic and non-amnestic subtypes.

	naMCI	aMCI	Statistics
Sample size (n)	206	197	
Age (mean, SD)	70.00 (9.45)	69.34 (8.67)	0.72 (1)
Sex (n, % woman)	135 (65.53)	117 (59.39)	1.62 (2)
Years of formal education (mean, SD)	11.44 (4.49)	10.84 (4.40)	1.35 (1)
MMSE (mean, SD)	28.60 (1.33)	27.78 (1.57)	5.66 (1) *
FACEmemory Total score (mean, SD)	62.84 (19.83)	50.08 (18.26)	6.71 (1) *

aMCI: amnestic mild cognitive impairment; naMCI: non-amnestic mild cognitive impairment; SD: standard deviation; MMSE: Mini-Mental State Examination. * *p*-value < 0.05.

**Table 4:** Metrics for the best-fitted model on the different feature sets.

Groups	Feature Set	Maximum BA for	Model	Balanced Accuracy	Sensitivity	Specificity
CH-MCI	Accumulated score	Long-term	Cutoff = 44.5	0.70 (0.05)	0.68 (0.06)	0.72 (0.06)
CH-MCI	Subscores	Long-term	RF	0.69 (0.06)	0.67 (0.08)	0.72 (0.08)
	Subscores + Demographics	Face Recognition	XGB	0.70 (0.06)	0.63 (0.14)	0.77 (0.08)
CH-aMCI	Accumulated score	Long-term	Cutoff = 44.5	0.76 (0.06)	0.81 (0.09)	0.72 (0.06)
	Subscores	Long-term	RF	0.76 (0.05)	0.80 (0.07)	0.73 (0.07)
	Subscores + Demographics	Long-term	RF	0.76 (0.06)	0.80 (0.08)	0.72 (0.08)
CH-naMCI	Accumulated score	Long-term	Cutoff = 42.5	0.64 (0.09)	0.52 (0.13)	0.76 (0.06)
	Subscores	Learning 1	RF	0.66 (0.02)	0.59 (0.05)	0.73 (0.07)
	Subscores + Demographics	Face Recognition	RF	0.67 (0.02)	0.60 (0.06)	0.74 (0.10)

BA: balanced accuracy; RF: random forest; XGB: XGBoost.

## Data Availability

The dataset generated and analyzed for this study will be made available by the corresponding author on reasonable request.

## Appendix A

Appendix A provides multiple regression analyses with each cognitive composite as the dependent variable and demographic features and FACEmemory subscores as independent variables.

**Table A1:** Multiple regression analysis with the attention cognitive composite as the dependent variable, and demographic features and FACEmemory subscores as independent variables.

Variable	Coefficient	z-Statistic	*p*-Value	95% CI
Years of formal education	0.045	6.32	<0.001	[0.031, 0.059]
Age	−0.005	−1.42	0.156	[−0.012, 0.002]
Sex ^1^	−0.066	−1.07	0.281	[−0.187, 0.054]
LN1	0.043	2.29	0.022	[0.006, 0.080]
LO1	−0.016	−1.00	0.316	[−0.049, 0.016]
LN2	−0.034	−1.38	0.168	[−0.083, 0.014]
LO2	0.033	1.87	0.061	[−0.002, 0.069]
RSN	−0.027	−0.90	0.367	[−0.086, 0.032]
RSO	−0.016	−0.83	0.405	[−0.056, 0.023]
FR	0.059	0.92	0.358	[−0.067, 0.185]
RLN	0.072	2.71	0.007	[0.020, 0.124]
RLO	0.011	0.62	0.535	[−0.024, 0.047]
REN	0.029	2.22	0.026	[0.004, 0.056]
REO	0.038	1.54	0.123	[−0.010, 0.088]

^1^ woman = 1 and man = 0; CI: confidence interval; LN1: names recalled in learning 1; LN2: names recalled in learning 2; LO1: occupations recalled in learning 1; LO2: occupations recalled in learning 2; RSN: names in short-term recall; RSO: occupations in short-term recall; RLN: names in long-term recall; RLO: occupations in long-term recall; FR: face recognition; REN: names correctly recognized; REO: occupations correctly recognized.

**Table A2:** Multiple regression analysis with the executive functions cognitive composite as the dependent variable and demographic features and FACEmemory subscores as independent variables.

Variable	Coefficient	z-Statistic	*p*-Value	95% CI
Years of formal education	0.041	7.98	<0.001	[0.031, 0.052]
Age	−0.009	−3.45	0.001	[−0.014, −0.004]
Sex ^1^	−0.046	−1.02	0.304	[−0.135, 0.042]
LN1	0.011	0.85	0.390	[−0.015, 0.039]
LO1	−0.008	−0.72	0.468	[−0.033, 0.015]
LN2	−0.001	−0.06	0.952	[−0.037, 0.034]
LO2	0.014	1.05	0.291	[−0.012, 0.040]
RSN	−0.025	−1.15	0.247	[−0.069, 0.018]
RSO	0.015	1.03	0.303	[−0.014, 0.044]
FR	0.003	0.07	0.941	[−0.089, 0.096]
RLN	0.040	2.08	0.037	[0.002, 0.078]
RLO	0.018	1.36	0.172	[−0.008, 0.044]
REN	0.035	3.64	<0.001	[0.017, 0.055]
REO	0.070	3.82	<0.001	[0.034, 0.106]

^1^ woman = 1 and man = 0; CI: confidence interval; LN1: names recalled in learning 1; LN2: names recalled in learning 2; LO1: occupations recalled in learning 1; LO2: occupations recalled in learning 2; RSN: names in short-term recall; RSO: occupations in short-term recall; RLN: names in long-term recall; RLO: occupations in long-term recall; FR: face recognition; REN: names correctly recognized; REO: occupations correctly recognized.

**Table A3:** Multiple regression analysis with the language cognitive composite as the dependent variable and demographic features and FACEmemory subscores as independent variables.

Variable	Coefficient	z-Statistic	*p*-Value	95% CI
Years of formal education	0.001	0.51	0.608	[−0.004, 0.007]
Age	−0.003	−2.65	0.008	[−0.007, −0.001]
Sex ^1^	−0.072	−2.97	0.003	[−0.119, −0.025]
LN1	−0.006	−0.86	0.385	[−0.021, 0.008]
LO1	−0.002	−0.34	0.729	[−0.015, 0.011]
LN2	−0.002	−0.20	0.840	[−0.021, 0.017]
LO2	0.006	0.85	0.392	[−0.008, 0.020]
RSN	−0.001	−0.13	0.893	[−0.025, 0.022]
RSO	0.010	1.34	0.178	[−0.005, 0.026]
FR	0.074	2.92	0.003	[0.024, 0.123]
RLN	0.010	1.01	0.312	[−0.010, 0.031]
RLO	−0.0006	−0.09	0.928	[−0.015, 0.013]
REN	0.015	2.85	0.004	[0.005, 0.025]
REO	0.017	1.81	0.069	[−0.001, 0.037]

^1^ woman = 1 and man = 0; CI: confidence interval; LN1: names recalled in learning 1; LN2: names recalled in learning 2; LO1: occupations recalled in learning 1; LO2: occupations recalled in learning 2; RSN: names in short-term recall; RSO: occupations in short-term recall; RLN: names in long-term recall; RLO: occupations in long-term recall; FR: face recognition; REN: names correctly recognized; REO: occupations correctly recognized.

**Table A4:** Multiple regression analysis with the memory cognitive composite as the dependent variable and demographic features and FACEmemory subscores as independent variables.

Variable	Coefficient	z-Statistic	*p*-Value	95% CI
Years of formal education	0.030	5.54	<0.001	[0.020, 0.042]
Age	−0.007	−2.64	0.008	[−0.013, −0.002]
Sex ^1^	0.168	3.50	<0.001	[0.074, 0.262]
LN1	−0.010	−0.72	0.467	[−0.039, 0.018]
LO1	0.004	0.36	0.716	[−0.021, 0.030]
LN2	0.045	2.34	0.019	[0.007, 0.083]
LO2	0.024	1.72	0.084	[−0.003, 0.052]
RSN	−0.021	−0.89	0.369	[−0.067, 0.025]
RSO	0.015	0.99	0.318	[−0.015, 0.046]
FR	−0.086	−1.73	0.082	[−0.185, 0.011]
RLN	0.050	2.42	0.015	[0.010, 0.091]
RLO	0.002	0.17	0.861	[−0.025, 0.030]
REN	0.019	1.82	0.069	[−0.001, 0.040]
REO	0.087	4.49	<0.001	[0.050, 0.126]

^1^ woman = 1 and man = 0; CI: confidence interval; LN1: names recalled in learning 1; LN2: names recalled in learning 2; LO1: occupations recalled in learning 1; LO2: occupations recalled in learning 2; RSN: names in short-term recall; RSO: occupations in short-term recall; RLN: names in long-term recall; RLO: occupations in long-term recall; FR: face recognition; REN: names correctly recognized; REO: occupations correctly recognized.

**Table A5:** Multiple regression analysis with the orientation cognitive composite as the dependent variable and demographic features and FACEmemory subscores as independent variables.

Variable	Coefficient	z-Statistic	*p*-Value	95% CI
Years of formal education	0.0004	0.24	0.806	[−0.003, 0.003]
Age	−0.0003	−0.33	0.740	[−0.002, 0.001]
Sex ^1^	0.0156	1.21	0.224	[−0.010, 0.041]
LN1	−0.0031	−0.78	0.430	[−0.011, 0.005]
LO1	0.0015	0.42	0.672	[−0.005, 0.008]
LN2	0.0027	0.52	0.599	[−0.007, 0.013]
LO2	0.0019	0.50	0.611	[−0.005, 0.009]
RSN	−0.0006	−0.10	0.918	[−0.013, 0.012]
RSO	−0.0037	−0.89	0.371	[−0.012, 0.004]
FR	−0.0291	−2.17	0.029	[−0.055, −0.003]
RLN	0.0000723	0.01	0.990	[−0.011, 0.011]
RLO	0.0046	1.22	0.222	[−0.003, 0.012]
REN	0.0021	0.73	0.460	[−0.003, 0.008]
REO	0.0253	4.84	<0.001	[0.015, 0.035]

^1^ woman = 1 and man = 0; CI: confidence interval; LN1: names recalled in learning 1; LN2: names recalled in learning 2; LO1: occupations recalled in learning 1; LO2: occupations recalled in learning 2; RSN: names in short-term recall; RSO: occupations in short-term recall; RLN: names in long-term recall; RLO: occupations in long-term recall; FR: face recognition; REN: names correctly recognized; REO: occupations correctly recognized.

**Table A6:** Multiple regression analysis with the praxis cognitive composite as the dependent variable and demographic features and FACEmemory subscores as independent variables.

Variable	Coefficient	z-Statistic	*p*-Value	95% CI
Years of formal education	0.012	3.50	<0.001	[0.006, 0.020]
Age	−0.009	−4.87	<0.001	[−0.013, −0.005]
Sex ^1^	−0.015	−0.49	0.621	[−0.078, 0.046]
LN1	−0.010	−1.07	0.284	[−0.029, 0.009]
LO1	0.002	0.29	0.768	[−0.014, 0.019]
LN2	0.018	1.45	0.146	[−0.006, 0.043]
LO2	−0.002	−0.29	0.765	[−0.021, 0.015]
RSN	−0.025	−1.64	0.101	[−0.056, 0.005]
RSO	0.004	0.41	0.675	[−0.016, 0.025]
FR	0.011	0.35	0.723	[−0.053, 0.076]
RLN	0.017	1.28	0.200	[−0.009, 0.044]
RLO	0.015	1.66	0.096	[−0.003, 0.034]
REN	0.009	1.41	0.159	[−0.004, 0.023]
REO	0.058	4.56	<0.001	[0.034, 0.084]

^1^ woman = 1 and man = 0; CI: confidence interval; LN1: names recalled in learning 1; LN2: names recalled in learning 2; LO1: occupations recalled in learning 1; LO2: occupations recalled in learning 2; RSN: names in short-term recall; RSO: occupations in short-term recall; RLN: names in long-term recall; RLO: occupations in long-term recall; FR: face recognition; REN: names correctly recognized; REO: occupations correctly recognized.

**Table A7:** Multiple regression analysis with the visuospatial/visuoperceptual cognitive composite as the dependent variable and demographic features and FACEmemory subscores as independent variables.

Variable	Coefficient	z-Statistic	*p*-Value	95% CI
Years of formal education	0.011	3.55	<0.001	[0.005, 0.018]
Age	−0.010	−5.95	<0.001	[−0.013, −0.007]
Sex ^1^	−0.050	−1.73	0.083	[−0.107, 0.006]
LN1	−0.010	−1.20	0.229	[−0.028, 0.007]
LO1	0.005	0.72	0.466	[−0.010, 0.021]
LN2	0.024	2.10	0.035	[0.002, 0.047]
LO2	0.006	0.81	0.417	[−0.010, 0.024]
RSN	−0.018	−1.27	0.202	[−0.046, 0.010]
RSO	−0.009	−1.00	0.315	[−0.028, 0.009]
FR	0.073	2.42	0.015	[0.014, 0.132]
RLN	0.007	0.56	0.572	[−0.017, 0.031]
RLO	0.011	1.30	0.190	[−0.006, 0.028]
REN	0.014	2.33	0.019	[0.002, 0.027]
REO	0.041	3.53	<0.001	[0.019, 0.065]

^1^ woman = 1 and man = 0; CI: confidence interval; LN1: names recalled in learning 1; LN2: names recalled in learning 2; LO1: occupations recalled in learning 1; LO2: occupations recalled in learning 2; RSN: names in short-term recall; RSO: occupations in short-term recall; RLN: names in long-term recall; RLO: occupations in long-term recall; FR: face recognition; REN: names correctly recognized; REO: occupations correctly recognized.

## Appendix B

Appendix B provides an overview of the hyperparameter search space used for the machine learning models.

**Table A8:** Hyperparameters of KNN.

Hyperparameter	Range
n_neighbors	[2, 100]
Weights	{uniform, distance}
Algorithm	{auto, ball_tree, kd_tree, brute}
leaf_size	[10, 100]
P	[1, 5]

**Table A9:** Hyperparameters of DT.

Hyperparameter	Range
max_depth	[2, 15]
min_samples_split	[0.01, 0.5]
min_samples_leaf	[0.01, 0.5]
max_features	[0.5, 1]
max_samples	[0.5, 1]
ccp_alpha	[1 × 10^−6^, 0.5]
Citerion	{gini, entropy}
class_weight	{balanced}

**Table A10:** Hyperparameters of SVM.

Hyperparameter	Range
C	[1 × 10^−5^, 1 × 10^2^]
Gamma	[1 × 10^−5^, 1 × 10^2^]
Kernel	{rbf}
class_weight	{balanced}

**Table A11:** Hyperparameters of RF.

Hyperparameter	Range
max_depth	[2, 15]
min_samples_split	[0.01, 0.2]
min_samples_leaf	[0.01, 0.2]
max_features	[0.5, 1]
max_samples	[0.5, 1]
ccp_alpha	[1 × 10^−6^, 0.5]
n_estimators	200
class_weight	{balanced_subsample}

**Table A12:** Hyperparameters of XGB.

Hyperparameter	Range
max_depth	[2, 15]
learning_rate	[1 × 10^−2^, 0.3]
Gamma	[1 × 10^−6^, 100]
min_child_weight	[0, 100]
subsample	[0.2, 1]
colsample_bytree	[0.2, 1]
colsample_bynode	[0.2, 1]
reg_alpha	[0.1, 10]
reg_lambda	[0.1, 10]
scale_pos_weight	[0.1, 10]
n_estimators	200
